# Gastroenterologist and surgeon perceptions of recommendations for optimal endoscopic localization of colorectal neoplasms

**DOI:** 10.1038/s41598-024-63753-x

**Published:** 2024-06-07

**Authors:** Garrett Johnson, Harminder Singh, Ramzi M. Helewa, Kathryn M. Sibley, Kristin A. Reynolds, Charbel El-Kefraoui, Malcolm B. Doupe

**Affiliations:** 1 Department of Surgery, Section of General Surgery, University of Manitoba, AE101-820 Sherbrook St, Winnipeg, MB R3A 1R9 Canada; 2 Clinician Investigator Program, University of Manitoba, Winnipeg, Canada; 3 Department of Internal Medicine, University of Manitoba, and CancerCare Manitoba Research Institute, Winnipeg, Canada; 4 Department of Community Health Sciences, Rady Faculty of Health Sciences, Max Rady College of Medicine, University of Manitoba, Winnipeg, Canada; 5 George and Fay Yee Centre for Healthcare Innovation, Winnipeg, MB Canada; 6 Departments of Psychology and Psychiatry, University of Manitoba, Winnipeg, Canada; 7 Manitoba Centre for Health Policy, Department of Community Health Sciences, Rady Faculty of Health Sciences, University of Manitoba, Winnipeg, Canada

**Keywords:** Colonoscopy, Colorectal cancer, Colorectal polyps, Endoscopy, Colonoscopy, Surgical oncology, Human behaviour, Colorectal cancer

## Abstract

National consensus recommendations have recently been developed to standardize colorectal tumour localization and documentation during colonoscopy. In this qualitative semi-structured interview study, we identified and contrast the perceived barriers and facilitators to using these new recommendations according to gastroenterologists and surgeons in a large central Canadian city. Interviews were analyzed according to the Consolidated Framework for Implementation Research (CFIR) through directed content analysis. Solutions were categorized using the Expert Recommendations for Implementing Change (ERIC) framework. Eleven gastroenterologists and ten surgeons participated. Both specialty groups felt that the new recommendations were clearly written, adequately addressed current care practice tensions, and offered a relative advantage versus existing practices. The new recommendations appeared appropriately complex, applicable to most participants, and could be trialed and adapted prior to full implementation. Major barriers included a lack of relevant external or internal organizational incentives, non-existing formal feedback processes, and a lack of individual familiarity with the evidence behind some recommendations. With application of the ERIC framework, common barriers could be addressed through accessing new funding, altering incentive structures, changing record systems, educational interventions, identifying champions, promoting adaptability, and employing audit/feedback processes. Future research is needed to test strategies for feasibility and effectiveness.

## Introduction

Colorectal cancer is one of the leading causes of cancer death in North America. Surgery, is the primary curative treatment for these cancers^1^. However, the colon can be resected in a variety of different configurations, therefore accurate preoperative localization is imperative to facilitate an effective operation. Colonoscopy, performed by an endoscopist (either a surgeon, or a gastroenterologist), is the gold standard for colorectal tumour localization^2^. However, colonoscopy is also a screening test, and therefore hundreds of colonoscopies are performed before a cancer is diagnosed^3^. Frequently the endoscopist is not the eventual operating surgeon^4^. Proper communication is therefore crucial to help surgeons make appropriate treatment decisions based upon information obtained during the initial colonoscopy. Unfortunately, there has been a wide variation in the documentation and communication of colonoscopy findings, including localization and characterization of colorectal cancers and precancerous tumours^5^. New recommendations to help standardize endoscopic tumour localization for colorectal cancers and polyps have recently been developed. These recommendations were established based upon comprehensive literature review and consensus between 23 Canadian experts^5^. Previously, endoscopists and surgeons have requested more standardized documentation and localization practices at endoscopy for colorectal tumours^6–9^. However, evidence suggests that creating new guidelines is insufficient to substantially impact clinician practices^10,11^. Meaningful practice changes are more likely when context-specific tailored strategies are used^12,13^. An important early step in implementation is to understand the perceptions of the end-users^14^. Our research objective was to identify the barriers and facilitators, as perceived by local gastroenterologists and surgeons, to using new recommendations designed to standardize endoscopic lesion localization for colorectal tumours. Findings from the present research can be used to help develop and pilot test contextualized interventions designed to improve adherence to the recommendations.

## Methods

### Overview and frameworks

This was a qualitative semi-structured interview study of gastroenterologists and surgeons in a single large central Canadian city (Winnipeg, Manitoba). We used directed content analysis methodology to explore participant perspectives on the new recommendations. This deductive approach to qualitative research is frequently used in health care implementation research and consists of describing phenomena according to participants’ perspectives in simple easily understood terminology. This methodology prioritizes fidelity to participants’ views, and compliments a pragmatic epistemology^15,16^. Pragmatism and postpositivism were the overlying approaches used to guide this research^17^.

The Consolidated Framework for Implementation Research (CFIR) was used to guide the creation and analyses of semi-structured interviews^13^. CFIR is an amalgamation of evidence-based implementation strategies across multiple disciplines, provides a comprehensive framework for assessing multiple aspects of implementation strategies, and has been used in multiple domains of health sciences research^18^. Guided by CFIR, data from surgeons and gastroenterologist interviews were analyzed separately to identify and compare the perceived facilitators and barriers to implementing the new documentation and tumour marking practices. Lastly, researchers have developed a new tool for matching expert-recommended interventions to barriers according to each CFIR category (CFIR-ERIC)^19–22^. Barriers identified in the present research were aligned with the CFIR-ERIC tool to identify possible solutions to overcome barriers to implementing the new documentation and tumour marking practices.

### Study setting

This study was conducted with gastroenterologists and surgeons in Winnipeg, Manitoba, Canada. Winnipeg is the largest urban centre (population 800,000 people) in the province of Manitoba (population 1.4 million people), has the only colorectal cancer referral site in this province, and treats 800–900 colorectal cancers annually^23,24^. All gastroenterologists and surgeons in Winnipeg operate under a single payer publicly funded “fee-for-service” billing model. Each of the six hospitals in Winnipeg has an outpatient endoscopy suite. There are two non-hospital-based endoscopy units. Three of the hospitals have operating rooms for surgeries requiring hospital admission. Winnipeg endoscopist practice patterns have been well-described as having high repeat preoperative endoscopy rates (29%)^6,25–27^ and suboptimal endoscopy report quality^25,28^.

### Study participants and sample

Study sample size was guided by the concept of *theoretical sufficiency*^29^. According to this principle, the research process concluded once all relevant categories from the CFIR framework had sufficient illustrative examples. Interview transcripts were analyzed soon after each interview, and mapped to the CFIR framework. Participants did not review their transcripts. Gastroenterologist and surgeon perspectives were analyzed separately, to ensure theoretical sufficiency was achieved in each group.

Participants in this research were selected purposively to obtain feedback from practitioners working in each endoscopy suite and operating room across the city, including diverse sub-specializations, practice patterns, and career stages. Initial recruitment emails were sent to every gastroenterologist and general surgeon in Winnipeg to ensure all eligible individuals felt invited and included. Follow-up emails were directed at specific groups who were under-represented after initial responses. Participant informed consent was obtained prior to each interview.

### Interview guide development and data collection

A semi-structured interview guide was developed (Additional file 1) based upon questions recommended by the CFIR authors and available on http://cfirguide.org/^13^. The questions were iteratively refined through meetings with the research team including a gastroenterologist, surgeon, knowledge translation expert, and an expert qualitative researcher and psychologist. Questions were piloted with two senior general surgery residents to assess interview flow, length, content clarity and appropriateness. Revisions were made following the pilot interviews, and pilot data were not integrated into the analysis.

CFIR authors recommend that implementation researchers identify which CFIR constructs they will assess in advance based on the relevancy to the study, rather than to attempt to assess every construct at once^13^. Four of the five CFIR domains aligned with the study objectives. Detailed rationale for which constructs were included are reported in Additional file 2.

Interviews were conducted by video teleconference (Zoom Video Communications, San José, CA), according to local COVID-19 pandemic-related restrictions. Participants were provided with a copy of the recommendations both prior to and at the beginning of the meeting. A previously published visual infographic tool was used to help participants understand and refer to the recommendations^5^. All interviews were audio-recorded and later transcribed by the primary analyst.

### Data analysis

#### Units of analysis

Data were categorized according to the CFIR constructs separately by provider specialty (i.e., gastroenterologist or surgeon). Within each group, constructs were subsequently categorized as facilitators or barriers according to coded perceptions. Findings from the two groups were then compared using a triangulation process to identify common and contrasting themes between specialties.

#### Data coding

Interview transcripts were imported into NVivo software for Mac (version 12.2.0; QSR International, Melbourne, Australia) for analysis. Coding was performed in duplicate independently by two researchers (GJ and CEK) using directed content analysis^30^. This deductive qualitative research approach is best used when an existing theory has previously been established to explain an observed phenomenon^30^, and has been used previously for analysis of qualitative interviews using the CFIR^31^. Following this approach, transcripts were coded using a predetermined codebook and inclusion criteria (Additional file 3)^13^.

Each transcript was first analyzed at the entire transcript level; these were reviewed repeatedly and coded deductively to CFIR constructs according to the codebook. Data were then reviewed at the level of each interview question to check for additional information that was missed during initial coding. After the entire coding process, both analysts met and created a single unifying codebook through consensus.

#### Construct relative priority

After interview transcripts were coded, participant perspectives were ranked according to whether a CFIR construct was perceived as a barrier or facilitator to implementing the new recommendations. Ranking criteria were adapted from previous work, and which have been used previously to differentiate high from low-performance implementation settings^32,33^. Ratings were performed separately by both analysts. Ranking criteria were based on level of agreement among study participants’ expressed views, language strength, and concrete examples used to emphasize responses (Additional file 4).

#### Validation strategies

Multiple strategies were used throughout the research process to ensure results’ validity including triangulation, reporting disconfirming evidence, dialogic engagement, and reflexivity^34^. Validation strategy details are listed in Additional file 5.

#### CFIR-ERIC intervention mapping

Researchers have recently developed and refined a tool to align CFIR constructs to the expert recommendations for implementing change (ERIC) framework^20,22^. Barriers identified in a study setting can be entered into the tool’s algorithm according to the CFIR framework, and subsequently the tool reports a prioritized list of strategies to consider, based upon prior consensus research^20,22^. The tool also reports the degree of consensus among the experts for each ERIC strategy as a method to address a particular CFIR barrier. According to this framework, strategies that were endorsed by ≥ 50% of the experts are deemed ‘Level 1’ strategies, and strategies that are endorsed by 20–49.9% of the experts are deemed ‘Level 2’ strategies^19–22^. The CFIR-ERIC authors suggest selecting a combination of both broadly applicable strategies, with high cumulative endorsement across multiple barrier constructs, in addition to specific strategies (i.e., level 1 strategy applicable to only one barrier)^22^. To ensure both approaches were addressed in this research, ERIC strategies were identified based on level 1 endorsement for each individual CFIR barrier, in addition to identifying more “general” strategies with high cumulative endorsement across all barrier constructs. ERIC strategies were stratified according to provider specialty.

Participants frequently proposed their own solutions during the interviews. In *post-hoc* analysis, these opinions were deductively coded to the ERIC framework and compared to those strategies identified via the CFIR-ERIC tool.

### Research team and reflexivity

The interviewer and primary analyst (GJ) was a male general surgery resident and master’s in science student throughout this research process. He was previously acquainted with many of the research participants prior to the interviews through his residency training. All attempts were made during the research to minimize the effect of biases these relationships may cause by acknowledging them throughout the research, discussing emerging findings with the research team, and critically examining the effects on the knowledge generated at each interview and during analysis.

### Ethics approval and consent to participate

This study was reviewed by the University of Manitoba Heath Research Ethics Board (HREB) for approval prior to data collection (reference number: HS25143, H2021:315). All experiments were performed in accordance with relevant guidelines and regulations. All participants provided informed consent prior to participation.

### Conference presentation

Partial results from this work were presented as a poster at Digestive Diseases Week 2022 in San Diego, CA and at the Canadian Surgery Forum 2022 in Toronto, ON, Canada.

## Results

### Participant demographics

There were 33 surgeons and 19 gastroenterologists who treat colorectal cancers identified as potential participants in Winnipeg during the study period. Of the 52 individuals invited, 11 gastroenterologists and 10 general surgeons participated in the study between October 2021 and January 2022. Participant demographics are shown in Table 1. Individuals participated from every endoscopy suite, hospital, and operating room in the city. Mean interview time was 56 min and 55 s.

**Table 1:** Participant characteristics (N = 21).

Clinical specialty	
Gastroenterologists	11
Academic	5
Community	6
Sub-specialization	
IBD	3
Therapeutic endoscopy	1
General surgeons	10
Academic	7
Community	3
Sub-specialization	
Colorectal	2
Surgical oncology	3
Clinical experience	
Most recent residency/fellowship training	
Canada	15
US	6
Colonoscopies performed per month*	
< 20	3
20–40	9
41–60	5
61–80	2
Colorectal cancer operations per month (surgeons only)	
0–1	4
2	4
3–4	2
≥ 5	0
Median years in practice**	10.5 (IQR: 3–21)
Mean age in years	46.8 (SD ± 11.1)
Gender (% female)	24

*Excluding two surgeons who do not routinely perform colonoscopy as part of their clinical practice.

**As an attending physician (excluding subspecialty training).

### CFIR content analysis

Twenty-seven CFIR constructs were assessed and deemed relevant to the research questions. Perceived barriers and facilitators to following the new recommendations are summarized according to construct relative priority rankings in Table 2.

**Table 2:** CFIR rankings stratified by participant specialty.

	Gastroenterology	Surgery
1. Intervention characteristics		
Intervention source	0	+ 1
Evidence strength and quality	− 1	− 1
Relative advantage	+ 1	** + 2**
Adaptability	+ 1	+ 1
Trialability	** + 2**	** + 2**
Complexity	+ 1	** + 2**
Design quality and packaging	+ 1	+ 1
Cost	− 1	− 1
2. Outer setting		
Patient needs and resources	0	0
Cosmopolitanism	** + 2**	+ 1
Peer pressure	0	0
External policy and incentives	− **2**	− **2**
3. Inner setting		
Structural characteristics	+ 1	** + 2**
Networks and communications	0	0
Culture	0	0
*Implementation climate*		
Tension for change	+ 1	+ 1
Compatibility	0	− 1
Relative priority	0	0
Organizational incentives and rewards	− **2**	− **2**
Goals and Feedback	− 1	− **2**
Learning climate	0	+ 1
* Readiness for implementation*		
Leadership engagement	+ 1	0
Available resources	− **2**	− **2**
Access to knowledge and information	− 1	− 1
4. Characteristics of individuals		
Knowledge and beliefs about the intervention	− 1	− 1
Self-efficacy	0	+ 1
Individual identification with organization	− 1	− 1

− 2 = “major” barriers, universally recognized as barriers by all participants with specific illustrative examples; − 1 = minor barriers, mixed opinions with overall barrier effect; ‘0’ = mixed perceptions; + 1 = minor facilitator, mixed opinions with overall enabling effect; + 2 = major facilitator, universally recognized as a facilitator by all participants, with specific illustrative examples.

Significant values are given in bold.

Both major (n = 4) and total facilitators (n = 11) were more numerous for surgeons compared to gastroenterologists (9 total, 2 major). Gastroenterologists and surgeons had eight net facilitator constructs in common: ‘relative advantage’ (major for surgeons only), ‘adaptability’, ‘trialability’ (major), ‘complexity’ (major for surgeons only), ‘design quality and packaging’, ‘cosmopolitanism (major for gastroenterologists only)’, ‘structural characteristics’ (major for surgeons only), and ‘tension for change’. Uniquely, surgeons identified ‘innovation source’, ‘self-efficacy’ and ‘leaning climate’ as facilitators, whereas gastroenterologists highlighted ‘leadership engagement’. The only universally acknowledged (major) facilitator for both groups was the ability of the recommendations to be trialed prior to full implementation.

Surgeons identified ten barriers whereas gastroenterologists identified nine. All nine gastroenterologist barriers were also identified as barriers for surgeons: ‘external policy and incentives (major)’, ‘organizational incentives and rewards (major)’, ‘available resources (major)’, ‘goals & feedback’ (major for gastroenterologists only), ‘access to knowledge & information’, ‘knowledge & beliefs about the intervention’, ‘self-efficacy’, ‘individual identification with the organization’, ‘evidence strength and quality’, and ‘costs’. The tenth barrier for surgeons, ‘compatibility’, had more mixed perspectives for gastroenterologists. Table 3 provides a summary of barriers and facilitators identified within each construct with exemplar quotations according to gastroenterologists and surgeons.

**Table 3:** CFIR barriers and facilitators to implementation of the new endoscopic lesion localization recommendations according to gastroenterologists and surgeons.

Constructs	Barriers and facilitators	Exemplar quotations
Intervention characteristics		
Intervention source	*Wanted local surgeon and organization endorsement* (Gastroenterologists only; barrier)	“Put [health authority’s name] or something on there, just so that people see it's kind of official and expected of people.” (Gastroenterologist 12) "If this was something that [Surgeon X] was like, do this every single time, I would do it every single time” (Gastroenterologist 14)
	Perceived as a joint gastroenterology and surgery initiative (Facilitator)	“[This is] a joint statement and that would help gain the respect of the community members” (Gastroenterologist 4) “Just the fact that you're doing it through both is kind of a good indicator of why this would probably work. Like it comes through both GI and surgery.” (Surgeon 10)
Evidence strength and quality	Unaware of evidence for some key recommendations (Barrier)	“When you raise the saline bleb, won't that make the tattoo stay in the lumen and not go through to the serosa?” (Gastroenterologist 4) “If there is data showing that it's an issue, people going in to find colon cancer, they can't find them because it's only been [tattooed] in one spot, you know, I can change what I do” (Gastroenterologist 6) “Why does [tattooing under the lesion] make it more difficult [to resect] endoscopically?” (Surgeon 3)
	*Willing to adopt practices simply because they were recommended.* (Facilitator)	“I think I'm actually going to try that [saline bleb] because I really have a hard time getting a good tattoo” (Gastroenterologist 11) “If that's something that'll help, I don't mind” (Gastroenterologist 6)
Relative advantage	Some alternate solutions were identified (Barrier)	“Get a CT scan, presumably the cancer is going to show up.” (Gastroenterologist 8) “I do like the movement in surgery and medicine in general towards doing things like black boxes where, you know, you have a general recording of any type of procedure that's done.” (Surgeon 9) “Send all of the tumors just to surgeons and send all the whatever to gastroenterologists, like diarrhea and IBS. That would be perfect.” (Surgeon 20) “Maybe if the surgeons trust the person who scoped them, then they don't repeat it” (Gastroenterologist 6)
	Most participants could not think of a preferable solution (Facilitator)	“I can’t think of anything else” (Gastroenterologist 5) “From a feasibility standpoint, I think this is probably the only reasonable way to do it” (Surgeon 9)
Adaptability	Some changes to the recommendations were suggested (Barrier)	“You know, the documentation part is a little wordy” (Gastroenterologist 6) “I probably would take out the ‘photographs for all but tiny, benign appearing polyps,’ because nobody's going to probably do that.” (Gastroenterologist 11) “For the rectum, you could say, relationship to anal verge or anorectal ring, relationship to rectal folds. You might want to do that for rectum.” (Surgeon 1)
	Recommendations could be easily adapted or would not need to be adapted at all (Facilitator)	“You wouldn't have to change the guidelines at all” (Gastroenterologist 2) “I don't think they would be difficult at all [to adapt]” (Gastroenterologist 13) “Everything that we're talking about here, is just minor changes in practice” (Surgeon 9)
Trialability	Implementation of the recommendations was viewed trialable locally (Facilitator)	“I think our city is small enough that it could be easily done in the three sites, or whatever number of sites that people are doing endoscopies.” (Gastroenterologist 4) “If you guys did [a pilot study] and you needed people to do it, I would.” (Gastroenterologist 17) “I'd put it up, tell everyone and then see what happens.” (Gastroenterologist 8)
	*Pilot trial viewed as unnecessary* (Surgeons only; facilitator)	“I would be comfortable adopting this without any type of significant pilot or anything” (Surgeon 9) “The recommended changes in the guideline are so simplistic and common-sense that I don't think a pilot study is likely needed” (Surgeon 7)
Complexity	*Recommended polyp classification systems are too difficult* (Gastroenterologists only; barrier)	“Paris is very cumbersome. I've tried to learn Paris. It's very hard to apply it” (Gastroenterologist 11) “Obviously Kudo, NICE and JNET are ones that come to mind and even the Paris classification there's some lack of familiarity, there.” (Gastroenterologist 2)
	Most recommended practices are simple (Facilitator)	“It's very simple and it looks good and it's easy for people to follow.” (Gastroenterologist 8) "I like them. I think they're good and they're simple to use and easy to follow." (Surgeon 21)
Design quality and packaging	Full-length manuscript too long to use (Barrier)	“You've got a lot of good information here, but it's twenty-four pages for someone to wade through. It’s a challenge for even the most dedicated person to digest” (Gastroenterologist 4)
	Excellent design quality of the infographic (Facilitator)	“I love how it's just laid out and it's simple and it's, you know, the indications, boom. How to do it, and then what kind of documentation you want. It's just, it's elegant the way it's laid out."(Surgeon 19) “It's very clear how it's all put. It's just got really neat pictures” (Gastroenterologist 19)
Cost	Takes longer to follow the recommendations, resulting in reduced income. (Barrier)	“This might be slightly more time consuming, and if you have a busy slate of six colons and you take an extra five minutes per colon, you know six times five is an extra 30 min. That's an extra colon, that's an extra few hundred bucks that you're potentially not getting in your pocket at the end of the day.” (Gastroenterologist 2) "If you're doing a procedure that takes 14 min and it takes four minutes for the nurses to draw up some saline, then it's just sort of an additional step.” (Surgeon 10)
	Surgeons get paid to do repeat scopes (Barrier)	“It is still a fee for service system and repeat procedures result in payment for surgeons” (Gastroenterologist 4) “I think there are some people who re-scope just for the billing code” (Surgeon 15)
	New recommendations had low perceived cost and add net value (Facilitator)	“The reality is our slates are such that they're set. So really there should be no disincentive for having a colonoscopy that takes you, you know, 10 more minutes.” (Gastroenterologist 13) “I think there's pros and cons from anything. The net benefit is much greater because you want to be providing best quality service to the patient.” (Gastroenterologist 2) "This isn't going to slow me down" (Surgeon 15)
Outer setting		
Patient needs and resources	Unaware that repeat endoscopy occurs (Barrier)	“I am very surprised, very surprised because, you know, I see the reports from the surgeons I refer to, and I don't recall, other than distal tumors, anyway, repeating the colonoscopy” (Gastroenterologist 6) “I'm not sure that I believe that all surgeons routinely repeat endoscopy or that surgeons frequently repeat endoscopy.” (Surgeon 7)
	Organization doesn’t understand endoscopy patient needs or prioritize QI (Barrier)	“[The health authority] is a disaster when it comes to research. They don't understand that research drives good clinical care” (Gastroenterologist 8) “I would like to see an administrative structure where they value clinicians who want to do quality improvement and they facilitate it rather than just be blind to it or let these processes continue, like you pulling your hair out.” (Surgeon 15)
	Individual participants had a good understanding of patient needs within their organization (Facilitator)	“No one wants to go through any more colonoscopies than they absolutely have to. Everyone knows the ardour of drinking 4L of PegLyte, even if you haven't done it personally, it's not fun.” (Gastroenterologist 2) “People have to have tests redone. And no big deal for us, but obviously a big deal for the person who has to take the prep and more so, you know, spend the next two weeks at home thinking, ‘Oh my God, they didn't do something right in me.’” (Gastroenterologist 11)
Cosmopolitanism	*Minimal networking related to endoscopy or colorectal cancer* (Surgeons only; barrier)	“Endoscopy and endoscopic markings? Bupkis. I don't network with anybody about this. I barely network with the people next door.” (Surgeon 3)
	Strong connections with external institutions (Facilitator)	“I usually attend one, pandemic notwithstanding, every year, actually, There's CDDW, and I would often participate in DDW as well.” (Gastroenterologist 2) “Most of the people who I talk to still are in Toronto or Montreal or Edmonton, places where I know surgeons who I personally worked with, who work in those places.” (Surgeon 7)
Peer pressure	Unaware of external institution efforts to improve colorectal lesions localization (Barrier)	“I don't know that we're necessarily going to be late adopters, but I don't know how many people make it their priority to be an early adopter either.” (Gastroenterologist 18) “If somebody else has done it and proven it reduces the repeat scopes by ‘X’-percentage, then that's a much easier sell. If not, it might be a little bit challenging” (Gastroenterologist 19) “Would it be valuable to us from the perspective of our reputation and admiration of our peers? I think the answer is probably not within the realm of surgery.” (Surgeon 7)
	*Valued being the first to adopt new recommendations.* (Surgeons only; facilitator)	“People would want to say, yeah, Winnipeg had the first people to do this because there's really there's no downside to it.” (Surgeon 16) “It makes it look like we're staying on top of things” (Surgeon 14)
	*Falling behind other organizations as a motivator to adopt the new recommendations* (Gastroenterologists only; facilitator)	“Winnipeg is behind the rest of the country.” (Gastroenterologist 17) “Certainly, being on the forefront of making those types of changes is certainly a good thing and would only be looked at favourably.” (Gastroenterologist 2)
**External policy and incentives**	No policies to encourage recommended practices (Barrier)	“I don't know what the incentive is for them to do it.” (Gastroenterologist 12) “Maybe this comes from my background, but we should be monitoring how people are doing, and there should maybe be some sort of punishment if people aren't following the rules.” (Surgeon 1) "I don't think there should be an incentive. If you say this is standard of care, you should follow it” (Gastroenterologist 19)
	Fee-for-service reinforces repeat endoscopy (Barrier)	"If we're paid for per scope right, then there's an incentive to be able to re-scope" (Surgeon 3) “Repeat procedures result in payment for surgeons”. (Gastroenterologist 4)
Inner setting		
Structural characteristics	*Staff turnover prevents sustained QI* (Gastroenterologists only; barrier)	“The nurses turnover, managers turnover, just the culture doesn’t change” (Gastroenterologist 5)
	Small, well integrated community with central organization (Facilitator)	“We have a nice relationship now, and not an antagonistic relationship that we had 15 to 20 years ago between surgeons and gastroenterologists.” (Gastroenterologist 8) “We're organized to some degree through central intake, even though it's mostly a booking system. Which allows for dissemination of information and application of standards” (Gastroenterologist 11) “Winnipeg's a small enough place, everybody goes, ‘Yeah, okay. You know, the next guy is doing it, the guy down the street is doing it. I know them both. I'll do it.’” (Surgeon 14)
Networks and communications	Some past endoscopy initiatives were poorly communicated. (Barrier)	“Some things just come out of nowhere. We started doing endoscopic timeouts recently, similar to surgical timeouts. People just started doing that one day without ever talking to anyone. This sounds great. I think this is important. There's lots of research literature, but I didn't realise we were starting this here.” (Gastroenterologist 2) "Some of it is just this random, somebody somewhere sends an email and I look at it go, OK, we're doing this now, right" (Surgeon 3)
	*Poor engagement with virtual rounds* (Surgeon only; barrier)	"My sense is that a lot of people log into these round sessions and perhaps check their emails and do whatever else on their phone in the virtual format." (Surgeon 7)
	Performance feedback is rare between providers (Barrier)	“We don't really get feedback unless it's from the surgeon that we refer to.” (Gastroenterologist 5)
	Strong collegial networks between providers, multiple venues of multidisciplinary communication identified (Facilitator)	“I'd say most of us go to the GI link rounds. And say at any GI link rounds, you're probably getting two thirds of the people in the city in terms of GI." (Gastroenterologist 18) "There is already cooperation on lots patient care and research related initiatives already so that certainly helps. You know, there's things like the journal club, et cetera, which the surgeons also attends." (Surgeon 21)
Culture	Individuals resistant to change. (Barrier)	“It tends to flow back to the way it was being done for years.” (Gastroenterologist 5) “I think some of the older endoscopists are, you're not going to change what they do. Or they think they already do it already, but they don't.” (Gastroenterologist 12) I think we have a bit of a culture of apathy or not wanting to have to do more work" (Surgeon 10)
	Organization doesn’t value or support endoscopy QI, stresses costs over quality. (Barrier)	“There have been lots of things that, you know, we wanted to change that we just hit a wall with the hospital” (Gastroenterologist 8) “It’s a glacial process that doesn't sit well with patients’ needs and providers’ desires” (Gastroenterologist 4) "I think that [leadership within the organization] value financial efficiency and optics" (Surgeon 7)
	Individuals believed the culture was a facilitator (Facilitator)	“I think culturally people are open to these types of concepts, at least academically.” (Gastroenterologist 2) “People want to be good, people want to improve, and people want to be doing what's appropriate.” (Gastroenterologist 11) "I think the specific culture associated with endoscopy will facilitate these guidelines being implemented." (Surgeon 3)
Implementation climate		
Tension for change	*Individuals already follow all the recommendations.* (Gastroenterologists only; barrier)	“I think anybody who's trained in the last five years is [doing this already]” (Gastroenterologist 8)
	Unaware that repeat endoscopy occurs (Barrier)	“I am very surprised, very surprised because, you know, I see the reports from the surgeons I refer to, and I don't recall, other than distal tumors, anyway, repeating the colonoscopy” (Gastroenterologist 6) “I'm not sure that I believe that all surgeons routinely repeat endoscopy or that surgeons frequently repeat endoscopy.” (Surgeon 7)
	Viewed implementation as important (Facilitator)	“It's really important work. Even I sometimes don't always do it to the exact standards that I want to, which I think is largely to the same sort of type of standards that you sort of outline here” (Gastroenterologist 2) “It's very, very worthwhile and very exciting” (Surgeon 14)
Compatibility	Organization does not value endoscopy QI. (Barrier)	“You have to have people in power that care about GI endoscopy, because even though it can lead to colorectal cancer, it's not CancerCare Manitoba, and it's not cardiac. So that's where all the dough is.” (Gastroenterologist 8)
	Recommended practices differ from current practices and equipment. (Barrier)	“The saline bleb thing I don't know about" (Gastroenterologist 18) “I don't routinely tattoo if it's a huge honking cancer” (Gastroenterologist 17) “I don't really have ScopeGuide" (Gastroenterologist 5)
	*Desire to continue to repeat scopes* (Surgeons only; barrier)	“Prior to surgery, if I didn't do the scope, or one of my surgical colleagues didn't do the scope, I do it. Like, I repeat it.” (Surgeon 20),
	Individuals’ value standardization and endoscopy QI (Facilitator)	“I think a guideline is great. I think the more stuff that we have standardized, the better” (Gastroenterologist 13) “Having everyone do things similarly and with a standardised format makes it a lot easier” (Surgeon 3)
Relative priority	Providers identified alternate priorities	“Before COVID, it wasn't a priority. Now with COVID, they will always say, well, once we get through COVID, we'll consider it” (Gastroenterologist 4) “Greater than 90% of my colon cancers come from either myself or [Gastroenterologist X], so, it is biased by its generation. So, do I have a huge bunch of issues with this? No.” (Surgeon 16)
	Recommendations viewed as high priority (Facilitator)	“In the last few months, I don't know about what others have told you, but in my practice, I'm finding way more malignancies than not” … “the timing would be perfect [for a solution].” (Gastroenterologist 19) “The ongoing COVID era and human resource crisis in the health care system probably values efficiency more than QI innovation. I think the way I would pitch that as a sales tactic is that minimizing repeat endoscopy is efficiency” (Surgeon 7)
**Organizational incentives**	No relevant organizational incentives identified (Barrier)	“There's no penalties if you don't follow it” (Gastroenterologist 8) "I don't think there's any incentive other than just to the patient," (Surgeon 1)
**Goals and feedback**	No formal relevant feedback processes (Barrier)	“There are some provincial audits, but we never hear about them” (Gastroenterologist 6) “Chances are [currently] they're only going to get feedback if it's egregious, right, which is pretty rare” (Surgeon 9)
	*Informal feedback from surgeons occurs* (Gastroenterologists only; facilitator)	“We don't really get feedback unless it's from the surgeon that we refer to and they say, you know, I didn't see the tattoo or where you tattooed was totally way off the site that you said it was. So that kind of feedback actually helps me personally.” (Gastroenterologist 5)
Learning climate	Organization leadership does not value or encourage endoscopy QI. (Barrier)	“There have been lots of things that, you know, we wanted to change that we just hit a wall with the hospital.” (Gastroenterologist 8) “If you go the route of saying to management, this is the new way of doing it, you're going to get stymied. You're going to get bogged down in the bureaucracy” (Surgeon 14)
	There is insufficient time for reflective thinking and evaluation (Barrier)	“I've tried to change things up, but you just have to be diligent with it and it's easy to get in a routine, and that's why people don't change because you're so against the clock all the time” (Gastroenterologist 12) "I think time is probably the main constraint” (Surgeon 21)
	Individuals felt empowered to institute change (Facilitator)	"If it's something as simple as where I put the tattoos, I don't need to involve anyone, I just start doing it” (Gastroenterologist 2) “[Surgeons are] going to the conferences and picking up the new techniques and just doing them” (Surgeon 14)
	Endoscopy leadership values and encourages QI (Facilitator)	“[The endoscopy lead] is very open if we need things or asking about certain equipment.” (Gastroenterologist 12)
Readiness for implementation		
Leadership engagement	Unable to identify endoscopy site leaders. (Barrier)	“I'm not even sure who all the site leaders are.” (Gastroenterologist 18) “I don't even know who the site leader for endoscopy at [my hospital] is" (Gastroenterologist 7)
	Organizational leadership viewed as a likely impediment to implementation (Barrier)	“I don't know if [the health authority] would care so much” (Gastroenterologist 12) “Anything through the [the health authority], it's, yeah, just stupid bureaucracy.” (Gastroenterologist 19) "I would like to see an administrative structure where they value clinicians who want to do quality improvement and they facilitate it rather than just be blind to it or let these processes continue," (Surgeon 15)
	Described actions they could reasonably foresee leadership taking to facilitate implementation (Facilitator)	“If let's say [the endoscopy lead] was to send out a memo saying, Oh, by the way, here's this new suggested way of doing tattooing and just attaching that same infographic sheet. That would be very useful. I think that catches my attention and I'll print it out and stick on my wall.” (Gastroenterologist 5) “I'm site lead at [one hospital] for surgery, and I guess by extension, endoscopy, and so I could certainly see, you know, bringing it up at a site meeting or, you know, sort of an endoscopy standards type meeting” (Surgeon 10)
**Available resources**	Resources were missing for full-scale implementation (Barrier)	“I don't really have ScopeGuide” (Gastroenterologist 5) I'm sure you've seen that trying to fax these photographs doesn't work” (Surgeon 3) “There's no one centralized place to go and say, ‘OK, we have the report’” (Gastroenterologist 17) “I don't have an EMR (electronic medical record) in my office” (Gastroenterologist 4)
Access to knowledge and information	Providers require resources, information and reminders that do not currently exist (Barrier)	“I think if you had like these little posters up in every Endoscopy room beside the computer where we're all doing our paperwork every day. You know, eventually it will get ingrained in someone's mind to do these things” (Gastroenterologist 18) “They just need to be reminded of the technical aspect.” (Gastroenterologist 4)
	No additional training needed for most providers (Facilitator)	“I think everything is already within the scope of practice. Yeah. No additional training.” (Gastroenterologist 19) "I would like to think that somebody who is an experienced endoscopist doesn't need too much other than maybe the sheet that you've got there, right" (Surgeon 3)
Characteristics of individuals		
Knowledge and beliefs about the Intervention	Providers minimized the importance of the recommendations they chose to ignore. (Barrier)	"The one thing I probably would not do is just start raising with saline." (Surgeon 10) “I don't routinely tattoo if it's a huge honking cancer. Usually I find even if I have tattooed it, they'll still go in and scope them.” (Gastroenterologist 17)
	*Desire to continue to repeat scopes* (Surgeons only; barrier)	“Basically, the only reason why I don't need to rescope this person is like if I get a referral from a colorectal surgeon. Because they have all the anatomical information that I care about.” (Surgeon 1)
	Positive perceptions of the recommendations and valued their implementation. (Facilitator)	"I think what you're doing is very worthwhile.” (Gastroenterologist 4) “I think a guideline is great. I think the more stuff that we have standardized, the better” (Gastroenterologist 13)
Self-efficacy	*Some providers had skill deficiencies* (Gastroenterologists only; barrier)	“Even now, in practice, I'm still learning this type of stuff. And so even being able to describe like where the polyp is, how would you describe this? What's the NICE classification and what is your approach to taking it off? We don't articulate those things,” (Gastroenterologist 17) “I have the hardest time injecting at an oblique angle” (Gastroenterologist 6) “I really have a hard time getting a good tattoo” (Gastroenterologist 11)
	Possessed the skills to follow most recommendations (Facilitator)	"I don't think this requires any particular skill set that people don't already possess" (Gastroenterologist 2) It's all pretty standard stuff, that I think all endoscopists can do" (Surgeon 16)
Individual identification with organization	Little identification with organizational goals. (Barrier)	“I don't think people really have a huge feeling about leadership anyways” (Gastroenterologist 13) “I'm here for the patient, so any patient that comes to contact me, including my colleagues, I'll stop for them and we'll take the time and we'll just have to suck it up as a medical institution. But the [health region] is basically saying, we can't” (Surgeon 20)
	*Some thought the organization unfairly prioritized other aspects of patient care over endoscopy (Gastroenterologists only; barrier)*	“It's just that it has to be a priority, right? And, you know, before COVID, it wasn't a priority” (Gastroenterologist 4) “You have to have people in power that care about GI endoscopy, because even though it can lead to colorectal cancer, it's not CancerCare Manitoba, and it's not cardiac. So that's where all the Dough is.” (Gastroenterologist 8)
	*Burnout impeding implementation (Surgeons only; barrier)*	“You have to look at, especially with COVID, you have to look at how mentally fatigued everyone is and how they just don't feel like you could not give people enough money to work harder. You can't” (Surgeon 20)
	High degree of identification with job role as a patient care provider. (Facilitator)	"This is about patient care and patient safety and that type of thing. I mean, I feel like nobody should have a problem with it” (Gastroenterologist 13) “You appeal to a doctor's desire to be good for his patients and for patients in general, and that's all you really need to do for incentives” (Surgeon 14)

*COVID* Coronavirus infectious disease 2019, *CT* computed tomography, *NICE* narrow band imaging international colorectal endoscopic, *QI* quality improvement.

Italics signify a perception that was unique to one specialty group. Major barriers are signified in bold.

### CFIR-ERIC strategy matching

**Figure 1:**
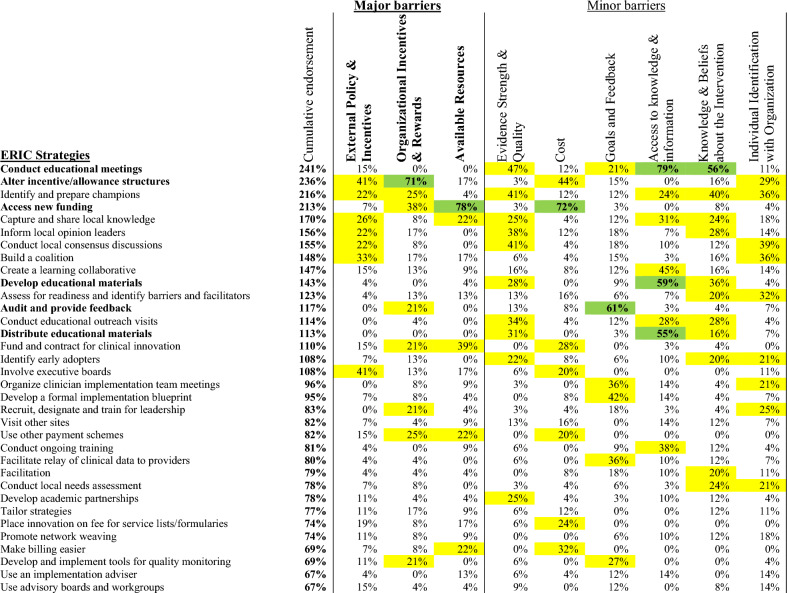

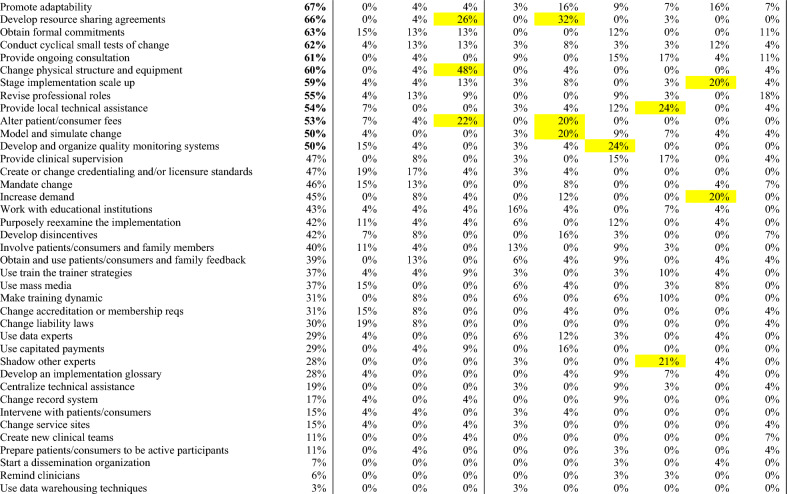
ERIC strategies matched to gastroenterologists’ CFIR barriers. Percentages indicate relative expert endorsement of a strategy to address a CFIR barrier according to CFIR-ERIC strategy matching by Waltz et al.^22^. Level 1 strategies (≥ 50% expert endorsement) displayed in green, and bolded in left column. Level 2 strategies (20–49% expert endorsement) displayed in yellow. Strategies are presented in descending order by cumulative endorsement across CFIR barrier constructs. Cumulative endorsement is the sum of expert endorsements for an ERIC strategy across all identified barriers. Major barrier constructs had universal agreement among participants, minor barriers had mixed perspectives.

**Figure 2:**
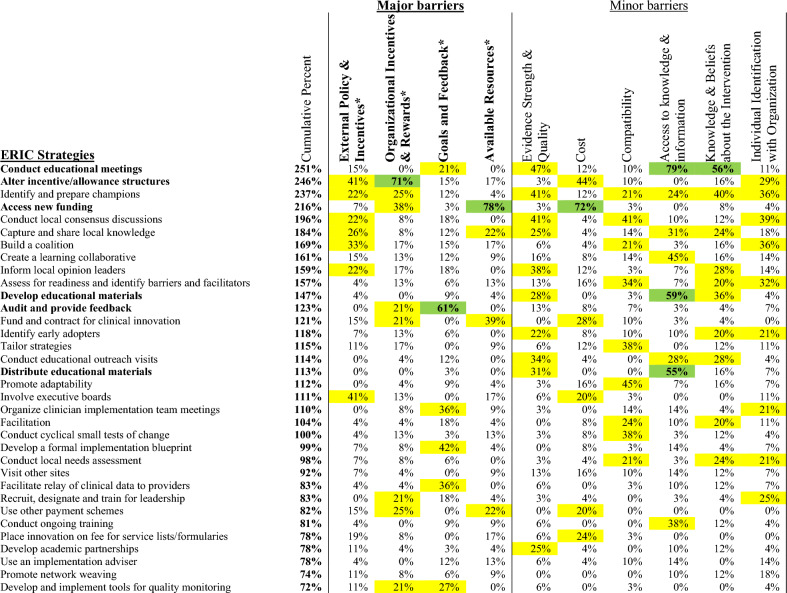

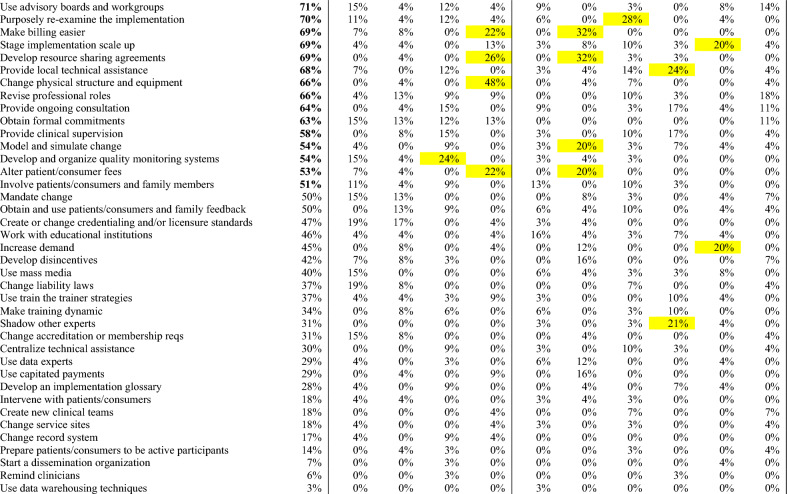
ERIC strategies matched to surgeons’ CFIR barriers. Percentages indicate relative expert endorsement of a strategy to address a CFIR barrier according to CFIR-ERIC strategy matching by Waltz et al.^22^. Level 1 strategies (≥ 50% expert endorsement) displayed in green, and bolded in left column. Level 2 strategies (20–49% expert endorsement) displayed in yellow. Strategies are presented in descending order by cumulative endorsement across CFIR barrier constructs. Cumulative endorsement is the sum of expert endorsements for an ERIC strategy across all identified barriers. Major barrier constructs had universal agreement among participants, minor barriers had mixed perspectives.

According to the CFIR-ERIC matching tool, strategies to address barriers identified by gastroenterologists and surgeons are displayed in Figs. 1 and 2, respectively. The top four ERIC strategies were identical for both gastroenterologists and surgeons: 1. ‘Conduct educational meetings’, 2. ‘Alter incentive/allowance structures’, 3. ‘Identify and prepare champions’, and 4. ‘Access new funding’. The CFIR-ERIC tool also identified six level 1 strategies (indicated in bold in the figures) to address CFIR barriers. Again, these strategies were identical for both gastroenterologists and surgeons: 1. ‘Conduct educational meetings’; 2. ‘Alter incentive/allowance structures’; 3. ‘Access new funding’; 4. ‘Develop educational materials’; 5. ‘Audit and provide feedback’; and 6. ‘Distribute educational materials’.

### Participant suggestions for implementation

Participants had many suggestions for how they would like to see the new recommendations implemented in their setting. Of the 73 total ERIC constructs, 24 were addressed by at least one participant during the interviews. The number of participants who endorsed a specific ERIC strategy are listed, and compared to percent endorsement according to the CFIR-ERIC strategy tool output in Table 4. The top five participant recommended strategies were: 1. ‘Audit and provide feedback’, “The best way probably would be for someone to have some degree of formalized feedback on their performance, which probably means receiving feedback on some scheduled time interval rather than feedback regarding each individual case.” (Surgeon 7); 2. ‘Change record systems’, “The only other debatable thing which I don't see it happening would be if somehow in EndoVault you actually recorded the endoscopies," (Gastroenterologist 18); 3. ‘Distribute educational materials’, “Place the infographic by the computer to reference during your paperwork.” (Gastroenterologist 18); 4. ‘Conduct educational meetings’, “A five-minute ad right before the next surgery or the next journal club or a five-minute plug before the next the GI Journal Club, right? Those are forums where you're getting enough people coming that you're going to get critical mass.” (Surgeon 14). 5. ‘Promote adaptability’: “Change anything that they perceive as an extra step”.

**Table 4:** Frequency of ERIC strategies suggested by interview participants compared to recommended strategies according to CFIR-ERIC strategy mapping.

ERIC strategy	Gastroenterologists			Surgeons		
	Endorsed	Opposed	CFIR-ERIC tool cumulative %	Endorsed	Opposed	CFIR-ERIC tool cumulative %
Audit and feedback*	11	0	117	9	0	123
Change record systems	9	0	17	8	0	17
Distribute educational materials*	10	0	113	7	0	113
Conduct educational meetings*^†^	7	0	241	7	1	251
Promote adaptability (modify guideline)	7	0	67	7	0	112
Stage implementation and scale up (pilot)	7	2	59	5	5	69
Inform local opinion leaders	9	0	156	3	0	159
Identify and prepare champions^**†**^	3	0	216	6	0	237
Mandate change	4	2	46	3	2	50
Develop disincentives	3	1	42	3	1	42
Involve executive boards	3	1	108	1	2	111
Alter incentive or allowance structures*^†^	2	1	236	2	0	246
Remind clinicians	4	0	6	0	0	6
Identify early adopters	1	0	108	2	0	118
Conduct ongoing training	2	0	81	1	0	81
Develop educational materials*	2	0	143	1	0	147
Change physical structure and equipment	1	0	60	1	0	66
Access new funding*^†^	1	0	213	1	0	216
Work with educational institutions	1	0	43	0	0	46
Obtain and use patient feedback	0	0	39	1	0	50
Capture and share local knowledge	1	0	170	0	0	184
Conduct educational outreach visits	1	0	114	0	0	114
Shadow other experts	1	0	28	0	0	31
Use train-the-trainer strategies	1	0	37	0	0	37

*Identified as a level 1 strategy to address barriers identified from CFIR-ERIC tool.

^**†**^Identified as a strategy with one of the top 4 highest cumulative endorsement percentages in the CFIR-ERIC tool.

## Discussion

Various groups have created recommendations to standardize lesion localization techniques^9,35–37^, however, there is large variation in these practices^25,27,38,39^. New Canadian Delphi consensus recommendations for optimal endoscopic localization of colorectal neoplasms provides a framework to standardize practices between providers^5^. Guided by the CFIR, the present research identifies across gastroenterologists and surgeons in a major Canadian city: (1) consensus on barriers and facilitators to implementing these new recommendations, and (2) areas with mixed perceptions both within and across study groups.

Importantly, most barriers (9 out of 10) identified were common to both gastroenterologists and surgeons. The CFIR-ERIC strategy-matching algorithm was used to propose externally validated (based upon expert consensus) types of strategies needed to overcome perceived barriers. Study participants also proposed their own implementation strategies. Due to similarities in perceived barriers between specialty groups, top ERIC strategies were identical for both specialties. There was also substantial overlap between expert-recommended strategies, and those suggested by our participants. Combining these approaches allows us to narrow down from a list of 73 ERIC categories into seven context-specific implementation strategies, including: 1. ‘Access new funding’, 2. ‘Altering incentives/allowance structures’, 3. ‘Change record systems’, 4. Educational interventions (i.e., ERIC recommends: ‘Distribute educational materials’, ‘Develop educational materials’ and ‘Conduct educational meetings’), 5. ‘Audit and provide feedback’, 6. ‘Identify and prepare champions’, and 7. ‘Promote adaptability’, The first three strategies in particular address the most common ‘major’ barriers identified by both specialty groups, which stem from a lack of internal and external organizational factors to incentivize compliance with the recommendations, and a lack of key resources needed to follow the recommended practices.

While one strength of the CFIR-ERIC framework is its flexibility, its breadth also makes these recommended strategies relatively non-specific. How these strategies can be utilized in future implementation efforts depends upon budget constraints, logistical considerations, and knowledge translation expert interpretation. Our participants’ suggestions allow us to tailor these recommended strategies into more prescriptive “next-steps,” recognizing that multiple interventions are possible, and these strategies need to be evaluated prospectively to ensure their validity.

One major barrier identified by both gastroenterologists and surgeons was a lack of specific resources required to follow the new recommendations. Therefore, ‘accessing new funding’, recommended by ERIC, is likely essential to any proposed solution. For example, new funding could be used to apply for resources such as modifications to the endoscopy medical record system or increase access to recommended materials (e.g., magnetic endoscope positioning device).

A lack of incentives to encourage compliance with the new recommendations was also a major barrier identified. Altering incentives, (e.g., pay-for-performance) is one of the most frequently studied ERIC strategies and is the subject of two recent systematic reviews. Both reviews identified mixed or inconsistent effects of pay-for-performance, and it is unclear which types of incentives targeted at which individuals are likely to lead to improved care^40,41^. While altering incentives is an expert-recommended strategy^20^, others suggest that this strategy is best used in combination with others, as it is unlikely to help overcome systemic barriers that prevent guideline adoption^42^. Altering incentive/allowance structures was also repeatedly mentioned as a desirable strategy by our participants. One popular suggestion was to provide additional compensation for a tattoo placed and documented exactly as recommended.

While not explicitly recommended according to the CFIR-ERIC strategy matching framework, ‘change record systems’ was a top strategy endorsed by our participants. Participants emphasized that local record systems do not allow for easy documentation of the recommended practices (e.g., tattoo information requires free text input). Furthermore, synoptic reporting has strong efficacy evidence for improved documentation of quality indicators in surgery^43,44^, diagnostic radiology^45^, and pathology^46^. Given the evidence of synoptic reports’ efficacy, changing medical records (i.e., implementing a purpose-specific synoptic report) represents an important strategy to consider locally, although would likely require additional financial resources to implement and maintain.

‘Educational interventions’ are designed to disseminate knowledge about the new recommendations. Educational interventions have been independently associated with increased clinician adherence to guidelines on a recent systematic review and meta-analysis^47^. However, optimal methods of clinician education to encourage guideline compliance are unknown^48^. Example strategies proposed by our participants include informational emails, infographic posters in the endoscopy suites, and grand rounds presentations. Combining educational interventions with additional implementation strategies appears to be superior to educational interventions alone in some settings^49,50^.

‘Audit and feedback’ has strong empirical evidence to support its’ effectiveness^20^, based upon a large Cochrane systematic review and meta-analysis^51^. Although the benefits of audit and feedback observed were generally small, and were highly dependent upon the method of feedback used and the baseline performance^51^. There are many potential aspects of the present recommendations to target for feedback. A common example raised by our participants was tattoo quality. Some endoscopists said they wouldn’t raise a saline bleb, place a 3-quadrant tattoo, or that the volume of injected ink was unimportant. However, previously local surgeons have raised tattoo quality as a major issue for lesion localization^38^. Prospective evaluation and feedback on these and other recommended practices is one method to address these concerns and provide real-world local data to encourage providers to fall in line with the recommendations.

‘Identify and prepare champions’ was a top recommended strategy, primarily as it is the highest endorsed ERIC strategy to address cultural barriers in an organization. Participants also mentioned champions as individuals who could continue to spur uptake of the recommendations on an ongoing basis after the initial implementation measures are over. As with many ERIC strategies, the effectiveness of implementation champions to address culture barriers are based primarily on expert opinion, and there is a paucity of evidence to inform the validity of this approach^20^.

Finally, ‘promote adaptability’ is important, as it reflects the reality that it is currently unknown if every recommendation must be followed to enhance localization and diminish repeat endoscopies, or if instead some recommendations can be ignored, and the desired effect will still occur^5^. To address this concept, the CFIR introduces the concept of an intervention’s “core components” versus its “adaptable periphery”^13^. The core components are the aspects of an intervention that must be followed for implementation success, whereas the adaptable components are the optional aspects that may not necessarily be required. The authors of the Delphi consensus recommendations suggest that recommendations with lower consensus could be considered “optional”, whereas those with higher consensus (i.e., consensus from the first Delphi voting round) are more strongly recommended^5^.

Taking all of these recommendations together, a possible implementation strategy in Winnipeg might include: modification of the endoscopy synoptic reporting system to include items from the new recommendations; purchase of magnetic positioning devices for all endoscopy suites in the city; the provision of additional compensation for a tattoo placed and documented exactly as recommended; a multi-faceted educational intervention including informational emails, infographic posters in the endoscopy suites, and grand rounds presentations; implementation of a systematic audit and feedback strategy targeted at tattoo quality, and compliance with recommended documentation; recruitment of individuals at each endoscopy suite to champion implementation of the new recommendations and maintain enthusiasm; and an adaptation plan where recommendations with lower consensus can be modified while maintaining the “core” highly recommended aspects.

## Contributions to the literature

To our knowledge, this is the first study in the literature to use the CFIR to examine barriers and enablers to implementing a new guideline targeted towards gastroenterologists and surgeons to reduce repeat endoscopy. Using this approach, we have applied modern implementation science methodology to identify strategies that may be used to enhance uptake of the recommendations in Winnipeg in the future. Others have attempted to evaluate endoscopy guideline implementation and quality improvement, however, those prior efforts are difficult to compare due to their lack of frameworks, and poor reporting of implementation strategies^25,38,52,53^. A strength of the current research is that by selecting robust, frequently used frameworks (CFIR and ERIC), we position the present research in the context of a broader body of literature^13,20^. This process has many benefits. For example, by following a structured theory-based framework, our research can serve as a sort of formula for others to follow suite. While our results are not necessarily applicable to implementation of the new recommendations outside of Winnipeg, the processes used are open to critique, and readily applicable elsewhere^54^. Our literature review also provides an up-to-date summary of the strengths and limitations of the CFIR and ERIC constructs evaluated. By using a framework, it is also imminently apparent to ourselves, and to other researchers, which aspects of the study setting have been evaluated, and which areas need further research (e.g., the entire ‘process’ domain, and the ‘innovation source’ and the ‘stage of change’ constructs). Had we used an inductive or tacit-knowledge-derived framework, deficient areas may not have been as apparent^54^.

Another major benefit of using an implementation science framework is that we have built upon the previous advances of others^13^. For example, our CFIR construct ranking criteria has been used previously on a post-hoc basis to examine factors associated with prior implementation success for weight management^33^ and hypertension strategies^32^. We built upon this prior research in multiple ways. First, we expanded up Damschroder and Lowery’s construct ranking system^32,33^. We modified their system and adapted to the pre-implementation phase, which has never been done previously. We propose using this ranking system as a new way to identify barriers significant enough to warrant selection of ERIC strategies. Previously researchers have selected ERIC strategies according to all CFIR barriers identified by participants, without a method of determining their relative significance^22^. Others selected ERIC strategies for all CFIR constructs, regardless of whether they were perceived as a barrier or facilitator^55^. Furthermore, now that we have a baseline assessment, we could also evaluate how perceptions of CFIR constructs in Winnipeg change in response to implementation strategies for our new recommendations.

Another unique aspect of our research is that we have expanded implementation science frameworks to a new discipline: endoscopy guideline-implementation. Colon cancer screening has been previously evaluated using implementation science framework^56–59^, including the CFIR^60,61^, but to our knowledge, guidelines for implementation of new endoscopy practices have not been evaluated with any implementation science framework.

A final unique aspect of our research is we have identified a disconnect between what strategies our clinician participants desire compared to those that are recommended by experts. To our knowledge, this is the first study to specifically examine the differences between strategies endorsed by the CFIR-ERIC experts, and those strategies desired by research participants. The CFIR-ERIC strategies are purported to address CFIR barriers according to expert opinion, but as discussed above, to date there is little empirical evidence to support selection of one strategy over another^22^. Comparison between ERIC strategies, or to those strategies identified by research participants represents an interesting avenue for further research. These comparisons may provide much-needed evidence for how a strategy can be selected in the future. Presumably, participants would be more likely to buy-in to ERIC strategies they specifically endorsed, although there is no evidence to support this yet.

## Limitations

Despite the important of our findings, this study design has some important limitations. First, the barriers and enablers identified are specific to the participants and settings evaluated, and should not be interpreted as broadly generalizable. This is not an inadequacy of the present research, rather, it is an inherent characteristic of qualitative descriptive research methodology^62^. Despite this limitation, the research processes used can be repeated in other settings to guide implementation elsewhere. Due to our use of CFIR, our findings may also be comparable to other settings.

A second limitation is that after our study completion, a new version of the CFIR was developed to reflect ongoing developments in knowledge translation research^63^. While the new CFIR has some new concepts, our data could be mapped to the new CFIR if necessary, to allow comparisons in future research.

Another limitation is that alternate coding systems or frameworks could have been used. There are hundreds of knowledge translation frameworks described^54^. We selected the CFIR and ERIC frameworks due to their broad applicability, and good fit for the research questions, methods, and settings. However, another framework could have been selected and possibly led to different results. Even within the CFIR, there are multiple methods of analysis and data coding that are possible^64,65^. For example, neither the CFIR nor the ERIC framework define what constitutes a significant barrier that is important enough to warrant application of dedicated implementation strategies^13,20^. In our analysis, we selected “net” barriers as those in need of ERIC strategies, with specific emphasis on ‘major’ barriers. However, there is no compelling evidence to suggest that only these barriers require solutions. In the present research, even net facilitator constructs had some associated barriers. One strategy is to target ERIC strategies to all barriers identified, no matter how infrequent^22^. Another approach is to target ERIC strategies to overcome barriers and also to amplify facilitators^55^. Had we followed either of these alternate approaches, we would have identified nearly every CFIR construct as in need of an ERIC strategy, which defeats the purpose of examining local barriers and facilitators a priori to identify targeted strategies.

One final limitation was that our research reflects only the perspectives of surgeons and gastroenterologists who participated. While a significant portion of all gastroenterologists and surgeons in Winnipeg chose to engage in the study (21 out of 52 possible participants), and hailed from diverse practice settings and backgrounds, our findings may not reflect the perceptions of those who chose not to participate. Furthermore, nurses, patients, healthcare administrators, allied health professionals, non-physician policy makers, and managers were excluded. This was done deliberately, as the new recommendations are targeted primarily at physicians, and their perspectives were felt to be key for devising next steps in the implementation process. Now that local gastroenterologist and surgeon perceptions are known, these additional stakeholders should be engaged to ensure their perspectives are considered for subsequent implementation.

## Conclusions

This research lays the groundwork for enhancing expert-recommended practices for colorectal lesion localization during colonoscopy in Winnipeg. We identified barriers and enablers from gastroenterologists and surgeons, mapping them to implementation science constructs. Despite some differences, both groups shared many perspectives, allowing us to create a unified list of implementation strategies to overcome barriers. We also compared participant-suggested strategies to those endorsed by implementation experts, forming a list of potentially effective local strategies. Future research should test these strategies' advantages and their impact on endoscopy quality.

### Supplementary Information

Supplementary Information 1.

Supplementary Information 2.

Supplementary Information 3.

Supplementary Information 4.

Supplementary Information 5.

## Data Availability

The datasets used and/or analysed during the current study are available from the corresponding author on reasonable request.

## Abbreviations

CFIR: Consolidated framework for implementation research
COVID-19: Coronavirus infections disease 2019
ERIC: Expert recommendations for implementing change
HREB: Health research ethics board
IQR: Interquartile range
NICE: Narrow band imaging international colorectal endoscopic
QI: Quality improvement
SD: Standard deviation
