# Supplementary material for: Gastroenterologist and surgeon perceptions of recommendations for optimal endoscopic localization of colorectal neoplasms
Source: Sci Rep. 2024 Jun 7;14:13157. doi: 10.1038/s41598-024-63753-x (PMC11161634; doi:10.1038/s41598-024-63753-x)
Supplement: Supplementary file 1 — Supplementary Information 1. [file 41598_2024_63753_MOESM1_ESM.docx]

**Focusing statement:**

Now we will move onto the meat of the interview. I will ask questions to get your perspectives on this new guideline. I want to emphasize that these questions are not a test for comprehension of the guideline, but are all designed to gather your perspectives so that both gastroenterologists and surgeons can use these recommendations most effectively in the future.

**Intervention characteristics** [additional probes: why/why not, can you give an example)

1. What are your first impressions of this guideline?
   - Do you think it is needed?
   - Do you agree with the recommendations?
   - Which ones don’t you agree with?
2. If you were referred a patient with a colon or rectal tumor who had a colonoscopy performed following all of the recommendations in this guideline, can you envision a scenario where you would need to repeat the scope?
   - Why? What more would you need?
3. How difficult do you think it would be to implement these recommendations in Winnipeg?
   - Why?
4. What do you perceive as the major barriers?
5. What resources would you need to overcome some of these barriers?
6. What are the major strengths, or advantages, we have in Winnipeg that might facilitate implementation of this guideline?
7. Do you think this guideline addresses a patient need?
   - How could the guideline be altered to better meet their needs?

**Relative Advantage**

1. Are you aware of any other interventions in Winnipeg that people have tried before to enhance colorectal lesion localization?
   - What advantages does the guideline have compared to those others?
   - What disadvantages?
2. Is there another solution to repeat endoscopy that you’d rather see implemented?

**Adaptability/Complexity**

1. What kinds of changes or alterations do you think need to be made to the guideline so it will work effectively in your setting?
   - Do you think these changes can be made? Why or why not?
2. Are there components that should not be altered?
   - Which ones?
3. How different are the guidelines’ recommendations from your current practice?
   - What about from your colleagues’ practices?

**Access to Knowledge & Information**

1. What kind of training would you need to be able to implement this guideline in your practice?
2. What kinds of information or materials about the guideline would you need?

**Trialability**

1. What are your thoughts on piloting a guideline implementation strategy in one of your endoscopy programs?
   - Do you think it would be realistic to pilot this guideline in your endoscopy suite?
   - Would a pilot study be important to you?

**Design Quality & Packaging**

1. Would you need a tool to help you use this guideline?
   - What types of tools or supports would you use?
   - (e.g., online resources, marketing materials, a toolkit, guideline summary, integration into EMR, posters on the wall, flow sheets, patient-oriented information, checklist)

**Cost**

1. Are you aware of any financial incentives or disincentives for following these guidelines?
2. Do you think it will cost you, in time or lost opportunity, to follow these guideline recommendations?
   - Do you view this as a net benefit or loss?
   - Why?

**---------------------------------------------------------------------------------------------------------------------**

**Questions about the settings:**

Changing gears a bit, the next set of questions pertain to the healthcare system that you work in, including the hospitals, clinics and health authority, and the individuals you work with.

**Networks, communication, implementation climate**

1. To what extent do you feel like you can try new things to improve your work processes?
   - Examples?
   - If you want to make a change, how do you get stuff done?
   - Who are your go-to people?
2. What role do site leaders play? What actions do they take?
   - Do you feel like quality improvement initiatives are valued or respected by leadership?
3. How do you typically find out about new information, such as new initiatives, or issues?
   - Is this effective? Is there another avenue that you would prefer?
4. Do you feel new endoscopy quality improvement initiatives should be initiated in Winnipeg?

**Goals and Feedback**

1. What do you think the general level of receptivity in your organization will be to implementing this guideline?
   1. Why/Where might barriers arise?
   2. How might this new guideline align with existing organizational goals?
2. Would you find it helpful to receive feedback on your work related to aspects of this guideline?
   1. [For example, feedback related to compliance with the guideline recommendations, your own repeat endoscopy rate, tattoo rate]
   2. What might be an acceptable method to receive feedback?

**External Policies & Incentives**

1. What (other) kinds of incentives would you support that could help ensure that the implementation of the guideline is successful?
   - (e.g., Hospital policies, mandates, pay for performance)
   - Are any of these incentives already in place?

**Culture** (general beliefs, values, assumptions that people embrace)

1. What role do you think your organization's culture will play in the implementation of this guideline?
   - Can you describe an example that highlights this?
   - Are new ideas/QI valued?

**Compatibility**

1. How could this guideline be integrated into existing work processes and practices?
   - How will it interact or conflict with current programs or processes?
   - What are likely issues that may arise?
2. Do you think this guideline’s implementation could replace or compliment a current program or process?
   - Which ones? In what ways?

**Leadership Engagement**

1. What would you want site leadership to do to help you use these guidelines?
   1. What types of barriers might they create?
2. Are there other influential individuals to get on board with implementation of this guideline?
   1. Who? Why?

**Peer Pressure/cosmopolitanism**

Past literature suggests that interaction between organizations, in particular “peer pressure”, can influence intervention success.

1. Do you know of any other organizations elsewhere that have looked at reducing repeat endoscopies?
   - [if yes] What have they done?
   - How does this affect support for implementing new strategies in Winnipeg?
2. Would implementing this guideline provide an advantage for our hospitals or health organizations compared to elsewhere?
   - Do you see any benefit to being ahead of other institutions?
3. To what extent do you network with other gastroenterologists and surgeons outside your own practice setting (i.e., people who you don’t work with clinically)?
   - What are the venues?
   - Do you attend local/national conferences?
   - Does your organization encourage you to network?

**Conclusion:**

1. Thinking back to how you answered these questions, were you thinking from the point of view of the endoscopist performing the initial scope, or from the perspective of the surgeon (or advanced endoscopist) receiving the endoscopy report, (or both)?
   1. Would your answers to the questions change thinking from the other perspective?
2. Are there any other aspects about this guideline that you wanted to mention, or that you feel I should have asked about?
