# Supplementary material for: Gastroenterologist and surgeon perceptions of recommendations for optimal endoscopic localization of colorectal neoplasms
Source: Sci Rep. 2024 Jun 7;14:13157. doi: 10.1038/s41598-024-63753-x (PMC11161634; doi:10.1038/s41598-024-63753-x)
Supplement: Supplementary file 2 — Supplementary Information 2. [file 41598_2024_63753_MOESM2_ESM.docx]

## Appendix 2. CFIR constructs and rationale for selection

(Adapted from Damschroder LJ, Aron DC, Keith RE, Kirsh SR, Alexander JA, Lowery JC. Fostering implementation of health services research findings into practice: a consolidated framework for advancing implementation science. Implementation Science. 2009 Aug 7;4(1):886–15)

| **Construct** | | **Examples from interview guide** | **If not included, rationale for exclusion** |
| --- | --- | --- | --- |
| **I. INTERVENTION CHARACTERISTICS** | | |  |
| A | Intervention Source | Not specifically assessed. These constructs are Included in general questions (e.g., Impressions of the guideline? Do you agree with the recommendations?) | We chose not to ask specifically their assessment of the evidence or perceptions of it being developed locally, as it is completely new, and we felt that participants will not have had the opportunity to assess these aspect in detail prior to the interview. However, should participants bring up these topics, they will be coded. |
| B | Evidence Strength & Quality |  |  |
| C | Relative Advantage | Included (e.g., Do you think it [the guideline] is needed? Are you aware of any other interventions in Winnipeg that people have tried) |  |
| D | Adaptability | Included (e.g., How difficult do you think it would be to implement in Winnipeg? What changes would you make?) |  |
| E | Trialability | Included (e.g., thoughts on piloting the guideline. How important is this to you?) |  |
| F | Complexity | What changes would you make? How do these recommendations differ from your current practice? |  |
| G | Design Quality & Packaging | Included (e.g., would you need a tool to help you use this guideline? What tool would you use) |  |
| H | Cost | Included (e.g. are you aware of any financial incentives?) |  |
| **II. OUTER SETTING** | |  |  |
| A | Patient Needs & Resources | Included. E.g., Do you think this guideline addresses a patient need? |  |
| B | Cosmopolitanism | Included. E.g., To what extent do you network? |  |
| C | Peer Pressure | Included. E.g., Do you see an advantage to being ahead of other institutions |  |
| D | External Policy & Incentives | Included e.g., are any incentives in place? |  |
| **III. INNER SETTING** | | |  |
| A | Structural Characteristics | This construct may be generally assessed through other questions probing barriers related to the setting. We have not included specific questions about the social architecture, age, maturity, and size of the organization. | The social architecture, age, maturity, and size of the organization is already generally known to us, and is often assessed objectively(18). Perceived effect of this construct was assessed in general terms. During piloting participant answers to the questions under this construct suggested on the cfirguide.org website overlapped with questions assessing implementation climate and design quality and packaging. These questions were thus removed for efficiency, but responses can be double coded to this construct if necessary, during analysis. |
| B | Networks & Communications | Included. E.g., how do you find out about new information? |  |
| C | Culture | Included e.g., what role do you think your organization’s culture will play? |  |
| D | Implementation Climate | |  |
| 1 | Tension for Change | Included e.g., Is this guideline needed in Winnipeg? |  |
| 2 | Compatibility | Included. E.g., How could this guideline be integrated into existing workflows? |  |
| 3 | Relative Priority | Included. E.g., Are there other interventions you’d rather see implemented? |  |
| 4 | Organizational Incentives & Rewards | Included. E.g., Questions about financial incentives and other incentives. Preferred incentives |  |
| 5 | Goals and Feedback | Included. Would you find it helpful to receive feedback on your work related to aspects of this guideline? |  |
| 6 | Learning Climate | Included. E.g., To what extent can you try new things? | Partially assessed. Questions about leadership double code to this construct. Gi and surgeon perceptions of value, and role in change process are not assessed, as these constructs were felt not to apply without a proposed implementation strategy in advance. |
| E | Readiness for Implementation | |  |
| 1 | Leadership Engagement | Included. E.g., How can site leadership help you use these guideline? |  |
| 2 | Available Resources | Included. E.g., What resources are available/do you still need to use these guidelines? |  |
| 3 | Access to Knowledge & Information | Included, but no specific questions. | The guideline is new, and has not yet been implemented in the organization, therefore questions about how this information is accessed are not yet applicable. |
| **IV. CHARACTERISTICS OF INDIVIDUALS** | | |  |
| A | Knowledge & Beliefs about the Intervention | Included. E.g., Is this intervention needed? Would you use this? | Questions about how individuals identify with the guideline and whether they can use it are included. As the guideline has not been implemented yet, questions pertaining to stage of change associated with guideline uptake were not developed. The cfirguide.org website does not provide suggested questions assessing Individual identification with their organization. Instead, this construct was generally assessed during the analysis when participants make applicable statements when responding to other questions. (Perceptions of provider burnout (included under individiaul identification with organization) are historically negatively associated with implementation success, but are beyond the scope of this current project and questions related to that issue were not included. |
| B | Self-efficacy | Included. E.g., Can you use this guideline? What barriers exist |  |
| C | Individual Stage of Change | Not assessed |  |
| D | Individual Identification with Organization | Included, but assessed only in part. Participants addressed this construct when asked about culture, leadership, and compatibility. |  |
| E | Other Personal Attributes | Excluded. | This construct is not part of the ERIC framework, and its role in identifying implementation strategies is unclear according to the chosen frameworks. Relevant characteristics of individuals that did not fit within the other constructs were coded here. |
