# Supplementary material for: Gastroenterologist and surgeon perceptions of recommendations for optimal endoscopic localization of colorectal neoplasms
Source: Sci Rep. 2024 Jun 7;14:13157. doi: 10.1038/s41598-024-63753-x (PMC11161634; doi:10.1038/s41598-024-63753-x)
Supplement: Supplementary file 3 — Supplementary Information 3. [file 41598_2024_63753_MOESM3_ESM.docx]

## Appendix 3. CFIR construct original short definitions and qualitative codebook inclusion criteria

(Adapted from Damschroder LJ, Aron DC, Keith RE, Kirsh SR, Alexander JA, Lowery JC. Fostering implementation of health services research findings into practice: a consolidated framework for advancing implementation science. Implementation Science. 2009 Aug 7;4(1):886–15)

| **Construct** | | **CFIR original short definition** | **Codebook definitions and inclusion criteria** |
| --- | --- | --- | --- |
| **I. INTERVENTION CHARACTERISTICS** | | | |
| A | Intervention Source | Perception of key stakeholders about whether the intervention is externally or internally developed. | Perceptions related to the entity that developed of the guideline/recommendations and/or where it was developed. Whether the guideline and/or the decision to implement is an “internal” organizational decision versus an “external” organizational decision is included in this construct. |
| B | Evidence Strength & Quality | Stakeholders’ perceptions of the quality and validity of evidence supporting the belief that the intervention will have desired outcomes. | Perceptions of the quality and validity of evidence supporting the belief that the recommendations will have desired outcomes (e.g., decreased repeat endoscopies, enhanced quality of Winnipeg endoscopy, enhanced care). There are two complementary components to this construct: 1. Perceptions related to recommendations as a whole; 2. Perceptions related to specific individual recommendations made. Both components will be examined in the analysis for an overall estimation of providers perceptions of the evidence strength & quality. |
| C | Relative Advantage | Stakeholders’ perception of the advantage of implementing the intervention versus an alternative solution. | Perceptions of the relative advantages and disadvantages of implementing the new guideline recommendations versus alternative solutions to repeat preoperative endoscopy and lesion localization errors in Winnipeg. Alternate solutions will be coded under this construct, but are not rated in barrier/facilitator analysis unless the participant feels the other solution is preferable. |
| D | Adaptability | The degree to which an intervention can be adapted, tailored, refined, or reinvented to meet local needs. | Perceptions of the degree to which the guideline recommendations, and their implementation can be adapted, tailored, refined, or reinvented to meet local needs. Suggestions for improvement are captured under this construct, but do not affect barrier/facilitator rating unless it is clear that the participant feels the change is needed but that the program cannot be adapted. |
| E | Trialability | The ability to test the intervention on a small scale in the organization, and to be able to reverse course (undo implementation) if warranted. | Perceptions on the ability to test the guideline recommendations on a small scale in their setting, and to be able to reverse course if warranted. Include statements about whether a pilot study is possible. Double code statement about whether a pilot study is needed, and possible desirable pilot study outcomes to evidence strength & quality. |
| F | Complexity | Perceived difficulty of implementation, reflected by duration, scope, radicalness, disruptiveness, centrality, and intricacy and number of steps required to implement. | Perceived complexity of the guideline and recommendations, reflected by duration, scope, radicalness, disruptiveness, centrality, and intricacy and number of steps required to implement. Exclude statements related only to how the guideline is presented/packaged and code to design quality & packaging below. |
| G | Design Quality & Packaging | Perceived excellence in how the intervention is bundled, presented, and assembled. | Perceived excellence in how the recommendations are bundled, presented, and assembled. There are two parts to this: 1. the full-length document; 2. the recommendations infographic. Participants were asked, although not required, to review both. Both components were equally weighted |
| H | Cost | Costs of the intervention and costs associated with implementing the intervention including investment, supply, and opportunity costs. | Perceptions related to costs from implementing and following the recommendations, including investment, supplies, and opportunity costs. Differentiate between costs associated with following/implementing the recommendations, versus statements relating to availability of existing resources (coded separately under available resources construct below). |
| **II. OUTER SETTING** | | | |
| A | Patient Needs & Resources | The extent to which patient needs, as well as barriers and facilitators to meet those needs, are accurately known and prioritized by the organization. | The extent to which patient needs, as well as barriers and facilitators to meeting those needs (relating to gastrointestinal endoscopy, colorectal cancer, and colorectal cancer surgery), are accurately known and prioritized by the organization.  There are two main parts included within this construct: 1. Statements demonstrating awareness of the needs and resources of those served by the organization (e.g., “the organization does or doesn’t understand the needs of patients because…”).  2. Statements that can be used to infer the level of awareness within the organization based on individual awareness (e.g., “I don’t know if this is an issue.”) |
| B | Cosmopolitanism | The degree to which an organization is networked with other external organizations. | The degree to which an organization is networked with other external organizations (e.g., conferences, formal and informal communication with individuals from outside organizations.) Exclude general networking and communication within the organization, and code those statements to the Networks & Communications construct in the Inner Setting domain (below). |
| C | Peer Pressure | Mimetic or competitive pressure to implement an intervention; typically because most or other key peer or competing organizations have already implemented or are in a bid for a competitive edge. | Perceived competitive pressure to implement a solution to repeat preoperative endoscopy. Include statements about perceived pressure or motivation from outside entities/organizations to implement the innovation, either because others have already addressed this (or similar) issue, or because of desire to be ahead of other organizations. |
| D | External Policy & Incentives | A broad construct that includes external strategies to spread interventions, including policy and regulations (governmental or other central entity), external mandates, recommendations and guidelines, pay-for-performance, collaboratives, and public or benchmark reporting. | Perceptions towards external strategies (i.e., government, licensing body, regulatory organizations) relating to the new recommendations and intended purpose of their implementation (reduced repeat endoscopy). Include statements regarding policies and regulations, external mandates, pay-for-performance, collaboratives, benchmark reporting, or even other recommendations and guidelines. Exclude statements related to policies/incentives from individual hospitals, clinics, Shared health, the WRHA, and code those instead to the Inner Setting construct “Organizational incentives”. |
| **III. INNER SETTING*** | | | |
| A | Structural Characteristics | The social architecture, age, maturity, and size of an organization. | Perceptions on how the social architecture, age, maturity, and size of the organizations within Winnipeg will affect implementation and use of the new endoscopy recommendations. Exclude perceptions related to outside hospitals (rural or other provinces) and code to cosmopolitanism or peer pressure, as appropriate. |
| B | Networks & Communications | The nature and quality of webs of social networks and the nature and quality of formal and informal communications within an organization. | Perceptions towards the nature and quality of webs of social networks and the nature and quality of formal and informal communications within the organizations in Winnipeg. Aspects related to communication specific to the new recommendations and the topic covered within are included within this construct, but also overlap with those in the “Goals & Feedback” construct (and are double coded). Exclude perceptions related to outside hospitals (rural or other provinces) and code to cosmopolitanism or peer pressure, as appropriate. Exclude perceptions towards resources required for communication for the purposes of patient care and code to “Available Resources” instead. |
| C | Culture | Norms, values, and basic assumptions of a given organization. | Perceptions of norms, values, and basic assumptions of the given organization, as they relate to endoscopy quality improvement, and implementation of past and future interventions within Winnipeg. There are two main ways used to evaluate this construct: 1. Statements directly addressing the culture within the organization (e.g., “I think the culture will be a barrier/facilitator because of X”)  2. Statements that can be used to infer the culture within the organization based on individual values (e.g., “This is not important to me and my peers.”). This second part overlaps with characteristics of individuals construct. |
| D | Implementation Climate | The absorptive capacity for change, shared receptivity of involved individuals to an intervention, and the extent to which use of that intervention will be rewarded, supported, and expected within their organization. | The absorptive capacity for change, shared receptivity of involved individuals to an intervention, and the extent to which use of that intervention will be rewarded, supported, and expected within their organization. This construct is an umbrella and automatically includes the following sub-constructs 1-6 below. |
| 1 | Tension for Change | The degree to which stakeholders perceive the current situation as intolerable or needing change. | Perceptions that the current situation (relating to topics covered in the recommendations) is intolerable or needing change. Unlike the other constructs, this is weighted as a barrier if participants are ambivalent to the current situation. |
| 2 | Compatibility | The degree of tangible fit between meaning and values attached to the intervention by involved individuals, how those align with individuals’ own norms, values, and perceived risks and needs, and how the intervention fits with existing workflows and systems. | Perceptions demonstrating the fit between meaning and values attached to the intervention (the new recommendations) by involved individuals and: 1. How those align with individuals’ own norms, values, and perceived risks and needs; 2. How those align with perceived organizational values (some overlap with culture); and 3. How the intervention fits with existing workflows and systems (overlap with “available needs and resources” construct). |
| 3 | Relative Priority | Individuals’ shared perception of the importance of the implementation within the organization. | Individuals’ shared perception of the importance of the implementation (of the new recommendation) within the organization. This construct has two components. 1. The relative priority of the intervention as individuals perceive it to be viewed from the organizations’ standpoint (e.g., “I do/don’t think Shared health will prioritize this”) 2. The relative priority of the intervention for individuals’, which can be used to infer the overall organization’s priorities (e.g., “This is my number one priority”).  Note, these criteria have substantial overlap with the constructs “Relative advantage” and “Tension for change”. The difference between this construct and the others is best illustrated by examples. E.g., someone may say the situation needs to change (tension for change facilitator), but they or the organization have other more important priorities (relative priority barrier). Individuals may say this is the best solution available (relative advantage facilitator), but this problem isn't important to them or the organization (tension for change barrier). |
| 4 | Organizational Incentives & Rewards | Extrinsic incentives such as goal-sharing awards, performance reviews, promotions, and raises in salary, and less tangible incentives such as increased stature or respect. | Perceptions towards extrinsic incentives (that are internal to the organization) such as goal-sharing awards, performance reviews, promotions, and raises in salary (separate from provincial fee-for-service reimbursement, which is classified in the outer setting), and less tangible incentives such as increased stature or respect from their peers within the organization (if related to external organizations coded to peer pressure construct instead).  Note: According to the CFIR-ERIC framework, this concept is rated as a barrier if there are no incentives, regardless of participants’ perceptions whether incentives are important to them for implementation. |
| 5 | Goals and Feedback | The degree to which goals are clearly communicated, acted upon, and fed back to staff, and alignment of that feedback with goals. | Perceptions that existing organizational goals (related to topics covered in the new recommendations regarding repeat endoscopy, adequacy of endoscopic lesion localization practices) are clearly communicated, acted upon, and fed back to staff, and alignment of that feedback with goals. Suggestions for improvement (i.e., type of feedback participants’ would like to receive) are coded under this construct. |
| 6 | Learning Climate | A climate in which: a) leaders express their own fallibility and need for team members’ assistance and input; b) team members feel that they are essential, valued, and knowledgeable partners in the change process; c) individuals feel psychologically safe to try new methods; and d) there is sufficient time and space for reflective thinking and evaluation. | This construct includes perceptions related to four components of the learning environment: 1. leaders express their own fallibility and need for team members’ assistance and input (some overlap with leadership engagement); 2. Team members feel that they are essential, valued, and knowledgeable partners in the change process 3. Individuals feel psychologically safe to try new methods; and 4. there is sufficient time and space for reflective thinking and evaluation (some overlap with available resources). |
| E | Readiness for Implementation | Tangible and immediate indicators of organizational commitment to its decision to implement an intervention. | Tangible and immediate indicators of organizational commitment to its decision to implement the new endoscopy recommendations. This construct is an umbrella that automatically includes the following sub-constructs 1-3 below. |
| 1 | Leadership Engagement | Commitment, involvement, and accountability of leaders and managers with the implementation. | General perceptions regarding the commitment, involvement, and accountability of leaders towards the implementation of the new recommendations in Winnipeg. As the recommendations are novel, with no proposed implementation strategy *per se,* included within this construct are general perceptions towards leadership and examples from past similar quality improvement initiatives in endoscopy/surgery. Leadership is assessed at multiple levels, (e.g., endoscopy site leads, endoscopy regional lead, nursing leadership, surgery leadership, organizational leadership) due to the complex nature of the system. For rating this construct, assessed are perceptions towards leadership within the Winnipeg organizational settings (i.e., exclude comments related to provincial political leadership, national licencing body leadership). Some participants had leadership roles within the organization. Their perceptions were weighted the same as general participants. |
| 2 | Available Resources | The level of resources dedicated for implementation and on-going operations, including money, training, education, physical space, and time. | Perceptions towards the level of resources available for implementation and on-going operation of the new recommendations, including money, training, education, physical space, and time. Coded under this construct are additional resources that are requested or required for use of the new recommendations. |
| 3 | Access to Knowledge & Information | Ease of access to digestible information and knowledge about the intervention and how to incorporate it into work tasks. | Awareness and perceptions of access to the new recommendations. Perceptions towards ease of access to digestible information and knowledge about the practices recommended by the new guidelines. |
| **IV. CHARACTERISTICS OF INDIVIDUALS** | | | |
| A | Knowledge & Beliefs about the Intervention | Individuals’ attitudes toward and value placed on the intervention as well as familiarity with facts, truths, and principles related to the intervention. | There are two parts to this construct, which are equally weighted and evaluated separately: 1. Individuals’ attitudes toward and value placed on the implementation of the new recommendations; 2. Familiarity with facts, truths, and principles related to the intervention (i.e., components of the guideline). This latter aspect has some overlap with the Evidence Strength & Quality construct. |
| B | Self-efficacy | Individual belief in their own capabilities to execute courses of action to achieve implementation goals. | Individual belief in their own capabilities to: 1. Follow all of the recommendations themselves; 2. Their perceptions of their colleagues’ ability to follow the recommendations; 3. Their ability to implement the recommendations; 4. Their perceptions of their colleagues’ ability to implement the recommendations. |
| D | Individual Identification with Organization | Characterization of the phase an individual is in, as he or she progresses toward skilled, enthusiastic, and sustained use of the intervention. | A broad construct related to how individuals perceive the organization, and their relationship and degree of commitment with that organization. There are multiple components: 1. Alignment between individual and organizational goals/values; 2. A perception of organizational justice; and 3. Providers' emotional exhaustion/burnout. |
| *Organizations assessed within the Inner Setting domain include local practice groups, individual hospitals, clinics, endoscopy suites, Shared health, and the Winnipeg regional health authority (WRHA); plus perceptions of the overall endoscopy organizational structure, and the surgery organizational structure (as they relate to colorectal tumors and their treatment) within Winnipeg. | | | |
