# Supplementary material for: Gastroenterologist and surgeon perceptions of recommendations for optimal endoscopic localization of colorectal neoplasms
Source: Sci Rep. 2024 Jun 7;14:13157. doi: 10.1038/s41598-024-63753-x (PMC11161634; doi:10.1038/s41598-024-63753-x)
Supplement: Supplementary file 4 — Supplementary Information 4. [file 41598_2024_63753_MOESM4_ESM.docx]

| **Criteria to assign ratings to constructs**  Adapted from Damschroder and Lowery^33^, and Muddu *et al*.^32^ | |
| --- | --- |
| **Rating** | **Criteria** |
| − 2 | The construct has a negative influence on the new guideline’s use. This construct has an impeding influence in work processes and/or an impeding influence in implementation efforts. Most interviewees (at least two) described with explicit examples how the key or all aspects of a construct manifests itself in a negative way. |
| − 1 | The construct has a negative influence on the new guideline’s use. This construct has an impeding influence in work processes and/or an impeding influence in implementation efforts. Interviewees make general statements about the construct manifesting in a negative way but without concrete examples: |
|  | • The construct is mentioned only in passing or at a high level without examples or evidence of actual, concrete descriptions of how that construct manifests |
|  | • There is a mixed effect of different aspects of the construct but with a general overall negative effect |
|  | • There is sufficient information to make an indirect inference about the generally negative influence and/or |
|  | • Judged as weakly negative by the absence of the construct |
| 0 | A construct has neutral influence on the new guideline’s use if: |
|  | • It appears to have neutral effect (purely descriptive) or is only mentioned generically without valence |
|  | • Interviewees from the same unit of analysis contradict each other |
|  | • Different aspects of the construct have positive influence while others have negative influence and overall the effect is neutral. |
| +1 | The construct has a positive influence on the new guideline’s use. This construct has an impeding influence in work processes and/or an impeding influence in implementation efforts. Interviewees make general statements about the construct manifesting in a positive way but without concrete examples: |
|  | • The construct is mentioned only in passing or at a high level without examples or evidence of actual, concrete descriptions of how that construct manifests |
|  | • There is a mixed effect of different aspects of the construct but with a general overall positive effect |
|  | • There is sufficient information to make an indirect inference about the generally positive influence |
| +2 | The construct has a positive influence on the new guideline’s use. This construct has a facilitating influence in work processes and/or a facilitating influence in implementation efforts. Most interviewees (at least two) described with explicit examples how the key or all aspects of a construct manifests itself in a positive way. |
