# Supplementary material for: Gastroenterologist and surgeon perceptions of recommendations for optimal endoscopic localization of colorectal neoplasms
Source: Sci Rep. 2024 Jun 7;14:13157. doi: 10.1038/s41598-024-63753-x (PMC11161634; doi:10.1038/s41598-024-63753-x)
Supplement: Supplementary file 5 — Supplementary Information 5. [file 41598_2024_63753_MOESM5_ESM.docx]

### *Validation strategies*

We used the concepts of triangulation, reporting disconfirming evidence, dialogic engagement, and reflexivity to guide this process.

The goals of triangulation are: 1. To achieve convergence between multiple sources of information to verify codes/categories; and 2. To create optimal conditions for challenging dominant perspectives, and to identify and report divergent opinions. There are multiple approaches to triangulation. In this study, we used the techniques of *methodological triangulation, data triangulation,* and *perspectival triangulation*^1^. As defined by Ravitch, *methodological triangulation* has two categories: *within-methods triangulation*, and *between-methods*. We used *within-methods triangulation*, in which one method was used for data collection (interviews) but had multiple ways of assessing constructs *within* the interviews. For example, multiple questions were included to assess different aspects of the same construct (e.g., to assess compatibility: “What would you change about this guide?” and “What parts are different from your current practice?”). Questions were also designed to elicit a mixture of perceptions (e.g., “What do you think are the barriers?”) and example narratives (“Can you give an example of a time culture affected a past implementation effort?”). In this way, constructs could be assessed at multiple points throughout the interview and compared. Occasionally, participants’ responses would reflect one perception (e.g., facilitator), but when probed further for examples or when asked a different way, their responses would change. Those instances, when they occurred, are highlighted in the presentation of the results.

*Data triangulation* is related to the concept of purposive sampling, and occurs when researchers attempt to attain data (relevant to the research question) from as many sources as possible. According to this concept, data were triangulated according to differing participant perspectives (*perspectival triangulation)*^1^. In reporting the results, multiple participant perspectives are illustrated with example quotes and attributed to their background (i.e., specialty) to demonstrate the diversity of the opinions expressed. This process is limited, however, in that only physician perspectives were sought for the present study.

Throughout the coding process, the validation strategy of *reporting disconfirming evidence* (i.e., discrepant information) was used to highlight the true diversity in personal experiences shared by participants. This was used both during the analysis for specific constructs, but also data were analyzed critically for entire cases (i.e., participants) whose opinions differed from the rest. As recommended by Ravitch, I also used these “outlier” perspectives to challenge my own preconceived or developing notions about the data, as well as my interpretation of the constructs that were developing^1^. For example, when I came across interviewees that disagreed with or challenged my understanding of the others (e.g., a construct was consistently a barrier and then a new participant said it was a facilitator), I deliberately went back to the previous transcripts to see if this perspective had been expressed before and I had missed it through my interpretation. I also reflected on reasons why this perspective might be different, which allowed me to identify important subgroups for further analysis.

*Dialogic engagement* was also used throughout the research design, data collection, and analysis process as a validation strategy. Dialogic engagement is a systematic processes for engaging in scheduled generative dialogue between the primary analyst and others, about the data collection, coding, and interpretation process^1^. According to this process, I met with members of the advisory committee at regular intervals during the research design, data collection and analysis process to check assumptions, pose questions, and challenge my interpretations to ensure they were fairly presented.

Finally, throughout the research process, I attempted to *engage reflexively* with the data and the participants by examining the effects of my implicit biases and positionality on the interpretation of the results (see critical reflexivity statement below). I kept a diary of my reflections, and carefully documented the rationale for all decisions made during the analysis process (i.e., decisions regarding which quotes to place in each construct, and how constructs were identified as barriers versus facilitators.)

*Participant validation* (i.e., member checking) was not a dominant validation strategy used in the current project. I had initially planned to perform a member check using the synthesized analyzed data and asking participants to comment by email. The benefits of this approach were the decreased time commitment for participants compared to other member check strategies, while also providing an opportunity to validate results by seeking disconfirming voices, and providing participants the opportunity for reflection on personal experiences and create opportunities to add data^2^. However, when I mentioned this aspect of the project to my participants at the end of the interviews, most were non-enthusiastic, and many suggested that they likely wouldn’t have time to respond meaningfully. Therefore, while I chose to send a summary of the study results to participants following study completion as a knowledge sharing strategy, none responded, so their comments were not used as part of the data analysis. Therefore, the only participant validation strategy I employed in this research was *integrated* within the interviews^1^. During our conversations, I would frequently synthesize my understanding of the information provided to me. This allowed for me to check my understanding of what the participants had expressed, and allowed participants an opportunity for reflection in case their words did not adequately reflect their intended meaning.

1. Ravitch S, Carl N. Validity: Processes, Strategies, and Considerations. In: Qualitative Research Bridging the conceptual, theoretical and methodological. SAGE publications inc.; 2015:186-211.

2. Birt L, Scott S, Cavers D, Campbell C, Walter F. Member Checking: A Tool to Enhance Trustworthiness or Merely a Nod to Validation? *Qual Health Res*. 2016;26(13):1802-1811. doi:10.1177/1049732316654870
